# 516 Negative Pressure Wound Therapy in Burns: A Prospective, Randomized-Controlled Trial

**DOI:** 10.1093/jbcr/irad045.113

**Published:** 2023-05-15

**Authors:** Christian Tapking, Jonathan Endlein, Jan Warszawski, Dimitra Kotsougiani-Fischer, Emre Gazyakan, Gabriel Hundeshagen, Sebastian Fischer, Christoph Hirche, Ulrich Kneser

**Affiliations:** BG Unfallklinik Ludwigshafen, University of Heidelberg, Ludwigshafen, Rheinland-Pfalz; BG Unfallklinik Ludwigshafen, University of Heidelberg, Ludwigshafen, Rheinland-Pfalz; BG Unfallklinik Ludwigshafen, University of Heidelberg, Ludwigshafen, Rheinland-Pfalz; BG Unfallklinik Ludwigshafen, University of Heidelberg, Ludwigshafen, Rheinland-Pfalz; BG Unfallklinik Ludwigshafen, University of Heidelberg, Ludwigshafen, Rheinland-Pfalz; BG Unfallklinik Ludwigshafen, University of Heidelberg, Ludwigshafen, Rheinland-Pfalz; BG Unfallklinik Ludwigshafen, University of Heidelberg, Ludwigshafen, Rheinland-Pfalz; BG Unfallklinik Ludwigshafen, University of Heidelberg, Ludwigshafen, Rheinland-Pfalz

## Abstract

**Introduction:**

Negative pressure wound therapy (NPWT) is a widely used tool for the treatment of complex wounds, including burns. However, there is only little data comparing NPWT to other wound dressings. This study aimed to evaluate the effectiveness of NPWT in small, acute burns of upper and lower extremities and to compare to the standard of care (SOC) at our institution.

**Methods:**

Patients that were admitted to our institution with burns on extremities between 0.5 and 10% of the total body surface area (%TBSA) burned were included and randomized to either NPWT or SOC (polyhexanide gel, fatty gauze, and cotton wool). Treatment was performed until complete wound healing. Patients that required skin grafts, received NPWT independent on the group. Patients pain level, time to wound healing, number of dressing changes and functional outcomes were assessed.

**Results:**

Sixty-two patients with burns, admitted between May 2019 and November 2021, were randomized into treatment with NPWT (n=32) or SOC (n=30). Both groups were similar regarding age (38.6±12.2 vs. 45.1±16.0 years, p=0.080), total burn size (2.95±2.42 vs. 3.31±2.93 %TBSA, p=0.595) and treated wound size (1.84±1.41 vs. 1.38±0.93 %TBSA, p=0.137). There were significant differences regarding healing time (11.19±4.92 vs. 8.70±3.58, p=0.027) and number of dressing changes throughout the study (2.19±1.49 vs. 3.97±1.69, p< 0.001). There were no differences in necessity of surgery (12.5% vs. 13.3%, p=0.329) pain assessments or functional outcomes.

**Conclusions:**

Healing time was longer in the NPWT, whereas NPWT group needed less dressing changes. This may be a psychological and logistical advantage. No differences were found regarding necessity of surgery pain and scarring.

**Applicability of Research to Practice:**

NPWT is safe in small burns and can lead to fewer dressing changes and pain.

